# Effectiveness of Osteopathic Manipulative Treatment in Adults with Irritable Bowel Syndrome: A Systematic Review and Meta-Analysis

**DOI:** 10.3390/healthcare11172442

**Published:** 2023-08-31

**Authors:** Francesca Buffone, Andrea Gianmaria Tarantino, Federico Belloni, Andrea Spadafora, Giorgio Bolzoni, Irene Bruini, Andrea Bergna, Luca Vismara

**Affiliations:** 1 Division of Paediatric, Manima Non-Profit Organization Social Assistance and Healthcare, 20125 Milan, Italy; francescabuffone.ost@gmail.com (F.B.); andreagtarantino@gmail.com (A.G.T.); 2 Principles and Practice of Clinical Research (PPCR), Harvard T.H. Chan School of Public Health–ECPE, Boston, MA 02115, USA; 3 Research Department, SOMA Istituto Osteopatia Milano, 20126 Milan, Italy; fede.belloni93@gmail.com (F.B.); spadafora.and@gmail.com (A.S.); bolzoni_g@libero.it (G.B.); andreabergna@soma-osteopatia.it (A.B.); 4 Intermediate Care Department, Caimi Hospital Foundation, 26019 Vailate, Italy; 5 Division of Neurology and Neurorehabilitation, IRCCS Istituto Auxologico Italiano, 28824 Piancavallo-Verbania, Italy; lucavisma@hotmail.com

**Keywords:** irritable bowel syndrome, gastrointestinal symptoms, osteopathic manipulative treatment, manual therapy, adults

## Abstract

The aim of this systematic review and meta-analysis was to evaluate the effectiveness of the osteopathic manipulative treatment (OMT) in adults with irritable bowel syndrome (IBS). A literature resview was carried out on the following databases: PubMed, Embase, Cochrane, Cinahl, Scopus, PEDro and ClinicalTrials.gov. 350 articles were recovered. Eligibility criteria were evaluated by two independent reviewers, including randomized controlled trials (RCTs), quasi-RCTs, or ongoing RCTs with OMT compared to any kind of control in patients diagnosed with IBS. Six studies (five RCTs and one ongoing RCT) were considered eligible. Four RCTs were classified as some concerns and one as high risk of bias. In the meta-analysis, OMT compared to sham/no intervention showed statistically significant results for abdominal pain (effect size ES = −1.14 [−1.66, −0.62]; *p* < 0.0001) and constipation (ES = −0.66 [−1.12, −0.20]; *p* = 0.005). Instead, OMT was not superior to the control for the IBS symptoms measured with the IBS Severity Score and the Likert scale (ES = −0.34 [−0.83, 0.16]; *p* = 0.19), and diarrhea (ES = −1.20 [−2.84, 0.43]; *p* = 0.15). The quality of evidence was “low” for IBS symptoms in general for abdominal pain and constipation, while it was judged as “very low” for diarrhea. OMT turns out to be safe in the treatment of IBS without major adverse effects. OMT may be effective in IBS patients, however the results must be interpreted carefully due to the low methodological quality of the studies.

## 1. Introduction

Irritable Bowel Syndrome (IBS) is a chronic functional gut-brain interaction disorder estimated to affect 1 out of 10 people worldwide [1], and characterized by abdominal pain or discomfort in relation with defecation. Diagnosed with the Rome Criteria IV [2], it can be classified in different subtypes depending on the patients’ stool characteristics (i.e., with diarrhea, constipation, mixed, and un-subtyped) [3], and it highly affects the quality of life with high rates of somatization, increasing psychological disorders, and absenteeism from work [4,5,6,7]. The individual burden is so important that patients are willing to take a median 1% risk to die for a 99% chance of getting cured [8]. Moreover, IBS also has an impact on the economy of the healthcare system. For instance, in Europe, the direct per capita cost due to IBS amounts to €3000 per year, and, looking at Italy alone, the total annual cost ranges from six to eight billion euros [9].

Therefore, it is of utter importance to develop effective treatment strategies and therapies which can address IBS in its entirety, including the abdominal pain, bloating, diarrhea, constipation, the sense of abdominal tightness, urination, and dysfunctions of the sexual sphere, and all the psychological consequences. However, to date, there is limited evidence regarding the efficacy, safety, and tolerability of the IBS therapies due to the heterogeneity of this disorder [10]. Furthermore, the patients’ responses vary from individual to individual even when they have similar symptoms [11], probably because of the incomplete knowledge regarding the IBS pathophysiological mechanisms [12,13]. Among the different interventions trying to address IBS, recently some studies [14,15,16,17,18,19] have investigated the contribution of osteopathic manipulative treatment (OMT), a manual approach which focuses on the diagnosis and treatment of somatic dysfunction (ME 93.0 in the ICD-11 coding tool). OMT, through specific techniques (tissue stretching, joint manipulation, muscle energy techniques, myofascial release, craniosacral treatment, and visceral manipulations), [19] has the main purposes of improving physiological functions and supporting the body’s homeostasis [16,17,18].

To date, there is a systematic review from 2014 assessing the effectiveness of OMT for IBS symptoms with promising results [20]; however, it was judged as low in the AMSTAR-2 quality assessment tool by a recent overview of systematic reviews [21]. There are another two systematic reviews from 2021, however, the focus was not only on OMT, as they included other types of interventions, such as physiotherapy, and acupuncture [22,23]. Hence, it was necessary to update the evidence regarding OMT and IBS, and nevertheless a meta-analysis was still missing, therefore this systematic review and meta-analysis aims to summarize the current evidence about the effectiveness of OMT in patients with IBS for the IBS symptoms.

## 2. Materials and Methods

### 2.1. Protocol Registration

This systematic review and meta-analysis were performed according to the PRISMA statement. The protocol of the review is available on PROSPERO under the registration number CRD42022318570 (https://www.crd.york.ac.uk/prospero/display_record.php?RecordID=318570, accessed on 16 April 2022) [24].

### 2.2. Search Strategy

A literature search was performed to evaluate the efficacy of OMT on abdominal pain levels and abnormal bowel movements in patients with IBS. The literature was searched up to May 2022 in PubMed, Embase, Cochrane, Cinahl, Scopus, and PEDro, including also ClinicalTrials.gov, and the grey literature.

The search strategy included terms such as: “irritable bowel syndrome”, “irritable colon”, “meteorism”, “constipation”, “defecation”, “osteopat*”, “osteopathic manipulation”, “osteopathic medicine”, “osteopathic treatment”, “myofascial treatment”, “myofascial therapy”, “myofascial release”, “myofascial technique”, “visceral manipulation*”, “cranial therap*”, “craniosacral therap*” and “musculoskeletal manipulation”. These terms were combined with each other in different methods depending on each database used (see Appendix A).

### 2.3. Eligibility Criteria

This review had the following inclusion criteria: randomized controlled trials (RCTs), quasi-RCTs, pilot RCTs, or ongoing RCTs assessing the effectiveness and/or efficacy of OMT in adults (>18 years old) diagnosed with IBS according to the Rome Criteria IV, compared to a control group (CG). The outcomes of interest had to be abdominal pain levels and abnormal bowel movements. Concerning OMT, as it has an intrinsic variability, OMT could be performed alone or combined with other therapies, with no restrictions of time, type of techniques, or frequency. The experimental group could not have other kinds of manual therapies such as physiotherapy or massage, however,, other types of intervention (i.e., sham, usual care, pharmacological therapy, etc.), or no intervention could be accepted for the CG. We considered eligible articles in English, Italian, French, and German.

### 2.4. Study Selection and Data Collection

The records were managed and screened using Rayyan QCRI software (https://www.rayyan.ai/, accessed on 5 May 2022) [25]. After removing the possible duplicates, two reviewers (F.B. and A.S.) performed a first blinded reading to screen titles and abstracts. Then, they performed a second blinded screening of potentially eligible studies by reading the full text. Finally, the two reviewers extracted key information (author, year, study design, objectives, outcomes, sample size, sample age, gender, IBS symptoms duration, type of experimental intervention, comparison, and main results) from the included texts. Any conflicts were resolved by a third reviewer (Fr.B.).

The six significant articles were produced in these countries of origin: 1 in Germany, 1 in The Netherlands and 4 in France.

### 2.5. Outcomes

The primary outcome for this review and meta-analysis was the severity of IBS measured as the abdominal pain intensity and the frequency of constipation or diarrhea. Secondary outcomes included biopsychosocial variables measured by the questionnaires about quality of life, psychological variables (anxiety, depression, etc.), use of drugs, patients’ satisfaction, and adverse events (AEs).

### 2.6. Assessment of Risk of Bias

The methodological quality was assessed by the Cochrane Risk of Bias tool 2.0 for the randomized trials (RoB2) [26]. Two authors judged the risk of bias (low, some concerns, and high) with the following domains: randomization process (domain 1.1, 1.2, and 1.3), deviation from the intended interventions (domain 2.1 to 2.7), missing outcome data (domain 3.1 to 3.4), measurement of the outcome (domain 4.1 to 4.5), and selection of reported results (domain 5.1 to 5.3). In case of discrepancy, a third reviewer contributed to resolving the disagreement.

### 2.7. Data Synthesis

The characteristics and findings of each study were summarized with the descriptive statistics. Proportion was used for categorical data, while mean and standard deviation were used for continuous data.

The meta-analysis was performed using “Review Manager 5.4” (The Nordic Cochrane Center, https://training.cochrane.org/online-learning/core-software-cochrane-reviews/revman; accessed on 4 August 2022). In order to perform the meta-analysis, at least two RCTs were needed. These had to investigate at least one of the specified outcomes, comparable in terms of PICO parameters. Standardized mean difference (SMD) with 95% CI was used, using a random effects model, due to the methodological heterogeneity. The interpretation of the effect size was as follows: “small” from 0.2 to 0.49, “moderate” from 0.5 to 0.79, and “large” if greater than 0.8. Heterogeneity was measured with the “I^2^ statistic”. Heterogeneity (I^2^) was considered of “no importance” (range 0–40%), “moderate” (range 30–60%), “substantial” (range 50–90%), or “considerable” (75% or above) [27].

The overall quality of evidence was assessed using the “Grading of Recommendations Assessment, Development, and Evaluation” (GRADE) criteria, according to the following five key domains: risk of bias, inconsistency, indirectness, imprecision, and publication bias. Hence, the reviewers downgraded the evidence from “high” to “very low”, accordingly [28].

## 3. Results

### 3.1. Studies Selection

**Figure 1:**
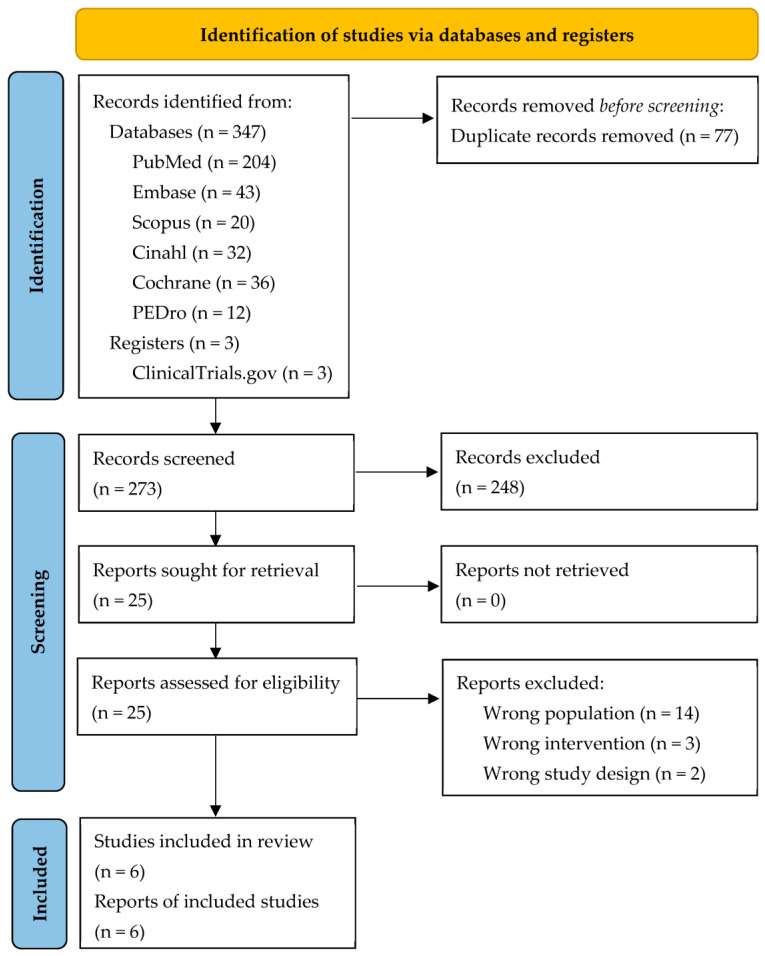
Flow diagram based on PRISMA statement.

The research identified 350 records. Before the screening, 77 studies were detected as duplicates and consequently removed. Hence, two reviewers (F.B. and A.S.) independently screened 273 records by reading the titles and abstracts, and 248 articles were excluded. They assessed 25 papers for eligibility by reading the full text. Finally, six studies were considered eligible and included in the review (Figure 1).

### 3.2. Description of the Studies

Five out of the six studies were RCTs (88.33%) [29,30,31,32,33], while one was an ongoing RCT (16.67%) [34]. Considering the study design, four trials and the ongoing study had a parallel design (88.33%) [29,30,31,33,34], while one study had a crossover design (16.67%) [32].

The primary outcome was the severity of the IBS symptoms in all the studies, measured with the IBS severity score in three studies (50%) [31,33,34], the visual analogue scale (VAS) in two studies (33.33%) [29,32], and the IBS quality of life questionnaire (IBSQOL) and Likert scale in one study (16.67%) [30]. The other three studies used IBSQOL as a secondary outcome [31,32,33]. Other secondary outcomes were the intensity and frequency of constipation and diarrhea, biopsychological variables, use of drugs, and AEs.

All the studies had the OMT as the experimental intervention, even though it differed for type of techniques, number of sessions, and frequency. One RCT performed individualized OMT (16.67%) [30], thus choosing different techniques according to each patient, whereas four RCTs provided semi-standardized OMT, which was planned by the ongoing RCT as well (83.33%) [29,31,32,33,34]. For the comparison, none of the studies had an active treatment: four RCTs provided sham OMT [29,31,32], which was also planned in the ongoing study [34], and two provided standard medical care [30,33]. The treatment period varied from a minimum of three weeks to a maximum of 13 weeks (7.17 ± 3.76), going from a minimum of two sessions to a maximum of five (3.5 ± 1.22). The frequency of interventions was considerably variable, since it went from a minimum of one treatment every two to three weeks, to a maximum of one treatment per week. Moreover, each session was either 45 or 60 min long (52.5 ± 8.66).

The total sample size in the five completed RCTs was 198 patients [29,30,31,32,33]; however, there was a dropout rate which ranged from 0% to 12%, with two studies not reporting the lost patients’ data even for the baseline characteristics [29,33]. Therefore, considering the dropouts, the total number of patients was 190, with 72% women and a mean age of 46.32 ± 3.46 years. Further details are shown in Table 1.

### 3.3. Risk of Bias

The risk of bias (RoB) was assessed in all the RCTs [29,30,31,32,33], except of the ongoing study [34]. In the RoB2, only one study (20%) [30] was judged to be at low RoB for the randomization process (domain 1), while three studies (60%) [29,31,32] were classified as some concerns. More specifically, Müller et al. [29] showed imbalances in the baseline characteristics, Attali et al. [32] did not provide any information about the allocation concealment and the baseline characteristics, and Florance et al. [31] about the randomization method and allocation concealment, although having a balanced group at baseline. Lastly, one study (20%) [33] was classified at high RoB since there was no reporting about the randomization method and the two groups showed an imbalanced group at baseline. Concerning the deviations from the intended intervention (domain 2), three studies (60%) [29,31,32] were judged at low RoB while two (40%) [30,33] were considered with some concerns because patients were aware of their assigned intervention. All the assessed trials had no significant missing outcome data, hence they were judged at a low RoB for this item (domain 3). For the measurement of the outcome (domain 4), the two trials with the unblinded participants (40%) [30,33] were classified as some concerns since they were their own assessors for the outcome “pain”. Lastly, for the selection and reported results (domain 5) all the trials (100%) were judged as some concerns since the reviewers could not find any report of a pre-specified analysis in the protocols or the trial registry records such as ClinicalTrials.gov.

**Figure 2:**
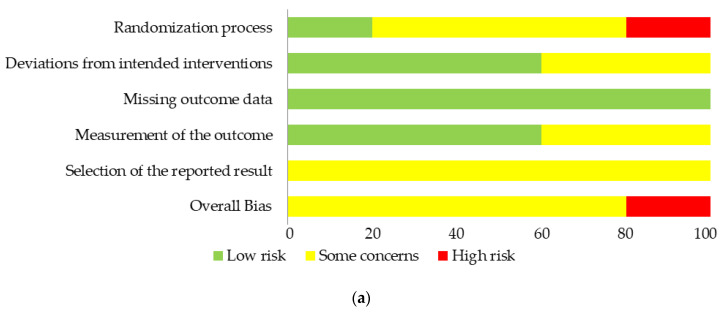

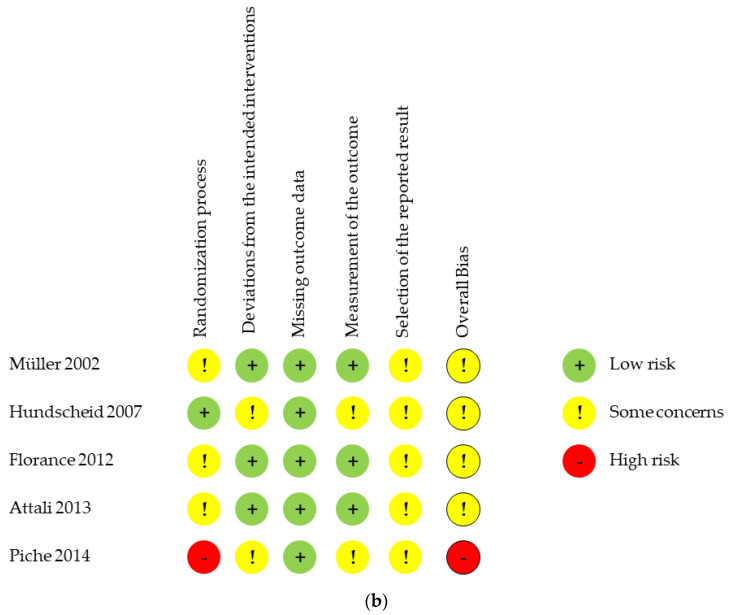
Risk of Bias Assessment graph of the included studies (**a**) summary of risk of bias presented as percentages across all included studies, (**b**) risk of bias for individual studies according to each domain [29,30,31,32,33].

Therefore, the overall RoB judgment revealed that four trials (80%) [26,27,28,29,30,31,32] were classified as some concerns, and one (20%) [33] at a high RoB. Results of the RoB2 assessment and judgment for each trial are summarized in Figure 2.

### 3.4. Description of the Main Results

The five completed RCTs showed statistically significant results for the severity of IBS [29,30,31,32,33]. Among them, two studies used VAS [29,32], including in Müller et al. [29] where after the last session there was a statistically significant reduction in abdominal pain for the OMT group (74.8%) as well as in the sham group (19.4%). For the between-group comparison after the last session, the pain reduction in the OMT group was significantly higher (*p* < 0.0001). Also, in Attali et al. [32] there was a statistically significant reduction in pain in both groups when compared to the baseline (OMT: 3.02 ± 0.59, *p* = 0.005; sham: 3.50 ± 0.54, *p* = 0.001), however there was no statistically significant difference in the between group comparison.

Two studies [31,33] used the IBS severity score, and had statistically significant results for the OMT group after the last session. Florance et al. [31] showed a 25.5% improvement in IBS symptoms for the OMT group (224 ± 102, *p* < 0.01), with no statistically significant difference in the between group comparison (*p* = 0.08). Instead, there was a statistically significant difference in favor of OMT compared to sham at day 7 (*p* = 0.01). The other study using the IBS severity score [33] showed a median reduction in symptoms of •39.5 (•60.9 to •9.2) with *p* = 0.05 in favor of the OMT group. Lastly, only one study [30] assessed IBS symptoms with the 5-point Likert scale showing statistically significant results in the between group comparison in favor of OMT (*p* = 0.02), however, there was no statistically significant changes in the intra-group analysis from the baseline to three months for the OMT group (9.1 ± 4 to 7.6 ± 4.5; *p* > 0.05) nor for the control group (8.7 ± 4 to 10 ± 4; *p* > 0.05).

Regarding the symptom of diarrhea, it was analyzed by four [29,30,31,32] out of five studies, and three had statistically significant results [29,30,32]. Müller et al. [29] showed improvements for the intensity of diarrhea after the last session (*p* = 0.0225) and at follow-up (*p* = 0.0165) in favor of the OMT group (decrease of 75.7%) compared to the sham group (decrease of 32.6%). Attali et al. [32] also showed statistically significant improvements for diarrhea in favor of the OMT group, specifically from the baseline to week five (*p* = 0.016), week 11 (*p* = 0.003), and at one-year follow-up (*p* = 0.029). In Hundscheid et al. [30], there were no statistically significant changes between the baseline and the further assessments, but at six months there was a statistically significant difference in favor of OMT (*p* = 0.02). Florance et al. [31] did not show any statistically significant results in either group.

Constipation was assessed in two out of five studies. In Müller et al. [29] there is a statistically significant result after the last session (*p* = 0.0183), as well as a decrease in constipation by 69.8% in the OMT group and by 17.2% in the sham group, with a statistically significant result at follow-up (*p* = 0.0080). In Attali et al. [32], there is a statistically significant difference between the baseline and week five (*p* = 0.022) and week 11 (*p* <0.001), but not in the follow-up after one year (*p* > 0.05).

Finally, three out of five studies assessed safety, and OMT was substantially safe [30,31,33]. In one study, there were no AEs at all [31] while in Hundscheid et al. [30] and Piche et al. [33], patients reported a slight increase in the severity of the symptoms after the first OMT session which resolved spontaneously, and a brief sensation of fatigue immediately after the intervention, respectively. Further details are shown in Table 2.

### 3.5. Effect of Interventions: Quantitative Synthesis

Four of the six studies comparing OMT with no intervention/sham treatment were included in the meta-analysis to assess the overall effect size (ES) for the IBS symptoms, abdominal pain, diarrhea, and constipation with regard to the outcome and type of variable [29,30,31,32]. One study [34] was excluded as it was an ongoing study, while another study [33]—although it was a completed RCT—was excluded as the data were presented with a median and interquartile range.

**Figure 3:**
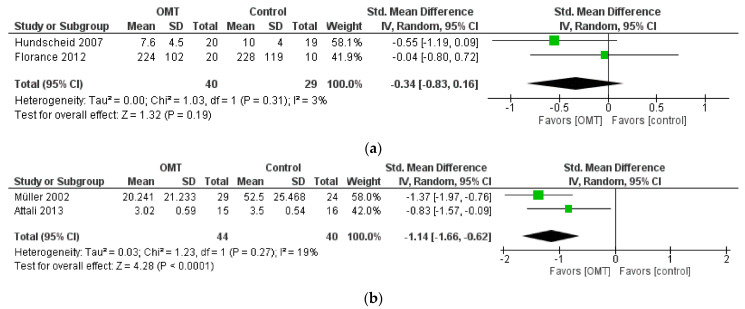

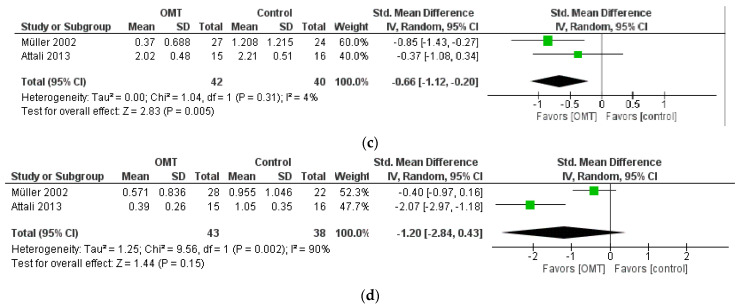
Forest Plot of (**a**) Likert Scale & IBS severity score, (**b**) VAS, (**c**) constipation, (**d**) diarrhea [29,30,31,32].

Regarding the IBS symptoms analyzed with the IBS Severity Score and the Likert scale, Neither study [30,31] reached statistical significance, thus showing no difference between the OMT and control (ES = −0.34 [−0.83, 0.16]; *p* = 0.19) groups. The heterogeneity was not important (I^2^ = 3%; *p* = 0.31) (see Figure 3a).

Concerning abdominal pain analyzed with VAS, both studies showed statistically significant effects in favor of OMT [29,32]. Hence, the overall effect (ES = −1.14 [−1.66, −0.62]; *p* < 0.0001) suggests that OMT should be considered superior compared to no intervention/sham treatment. The heterogeneity was not important (I^2^ = 19%; *p* = 0.27) (see Figure 3b).

Similarly, these two studies [29,32] have statistically significant results for constipation with an overall effect of (ES = −0.66 [−1.12, −0.20] *p* = 0.005), and a non-important heterogeneity (I^2^ = 4%; *p* = 0.31), suggesting that OMT might be superior compared to no intervention/sham (see Figure 3c).

For diarrhea, even though it was assessed by the four studies included in the meta-analysis, only two of them [29,30,31,32] reported the data required for the calculation of the effect size. The overall effect (ES = −1.20 [−2.84, 0.43] *p* = 0.15) suggested that OMT should not be considered superior to no intervention/sham treatment, and the heterogeneity was substantial (I^2^ = 90%; *p* = 0.002) (see Figure 3d).

The level of evidence was rated as “low” for IBS symptoms, abdominal pain, and constipation, however, it was considered as “very low” for diarrhea (see Table 3 for further details).

## 4. Discussion

To our knowledge, this is the first meta-analysis that evaluates the efficacy of OMT for the severity of IBS symptoms. In fact, although there are a previous systematic reviews assessing the effectiveness of OMT for IBS symptoms [20] and an overview of the systematic reviews summarizing the evidence of OMT in general [21], until now, there has not been an estimation of the effect size. Hence, we deemed it necessary to update the current evidence.

The results of this systematic review and meta-analysis showed that OMT compared to no intervention/sham treatment improved abdominal pain and constipation, while for diarrhea and IBS symptoms in general, there was no difference between the groups. Indeed, the number of studies was low, and in the quantitative synthesis, they were analyzed in couples due to the different kinds of variables or the lack of reported data, thus consequently reducing the number in the analysis. Hence, it would be of high interest having a larger number of studies with more homogeneous outcomes and better reporting. Moreover, although the pooled results for abdominal pain and constipation, and the results of the single studies alone were statistically significant, they have to be analyzed considering their methodological quality. As shown in the RoB assessment, four out of five studies were classified as “some concerns” [29,30,31,32] and the other one was judged at a high risk of bias [33]. The main issues regarded randomization and allocation concealment, which lacked clear reporting, blinding of patients, which did not occur in two studies even if the main outcome was a patient-reported endpoint, and the lack of any report of a pre-specified analysis in the protocols or trial registry records. A similar result was obtained by Bagagiolo et al. [21] where they judged all the studies at high RoB, whereas it disagrees with the RoB assessment by Müller et al. [20] where the methodological quality was considered high—hence with low RoB—for all the included studies. Interestingly, the previous analyses were performed with a very similar RoB assessment tool to the Cochrane Back Review Group [35].

In the analyzed studies, OMT was substantially safe, with none or mild to moderate transient AEs, and it showed promising results, which should encourage the researchers to develop further studies necessary to guide clinicians. Considering the low invasiveness of OMT, and its effects on the autonomous nervous system and pain [36,37,38,39], there is a biological rational that could justify OMT as an adjunctive therapy for IBS which is considered a stress-sensitive disorder. In fact, evidence showed that a condition of chronic stress leads to alteration in the intestinal microbiota—thus affecting the immune and nervous systems—and at the same time, it influences the hypothalamic-pituitary-adrenal axis with the subsequent secretion of cortisol, adrenocorticotropic and corticotropin-releasing hormones which affect gut function [39].

To date, there is no gold standard for this syndrome, and IBS patients are mainly treated with antispasmodic drugs and dietary changes [13]. Introducing a non-invasive complementary approach influencing the visceral system and the gut-nervous system interaction, such as OMT, would benefit this population [40].

Moreover, it seems that OMT improves chronic disorders [41] and has a positive influence on brain activity, decreasing inflammation and central sensitization [42,43,44,45]. The biological rationale of the osteopathic manipulation and its influence on the fascial component suggest that OMT could reduce abdominal pain and improve constipation.

However, without an acceptable number of high methodological studies, the role of OMT as an adjunctive therapy for IBS patients remains a mere speculation. Therefore, we strongly recommend new high-quality studies planned and performed according to the evidence-based guidelines [46,47]. Indeed, manual therapies have to deal with some issues not encountered by other interventions—for instance the lack of blinding for the operator—and OMT itself also has to face the intrinsic variability of its techniques, but this one limit should not prevent it from improving other methodological aspects and running high quality clinical trials. Another aspect that could help improve the evidence of the efficacy of OMT could be a specific assessment of the somatic dysfunction according to the variability model. In fact, it is suggested that the assessment of the somatic dysfunction could be more reliable if performed within the neutral zone [16,48]. A more specific oriented and standardized evaluation would improve the selection of the structures requiring the osteopathic treatment, thus leading to better results.

### 4.1. Quality of Evidence

The quality of evidence (see Table 3) was judged as “low” for IBS symptoms, abdominal pain, and constipation, while it was considered as “very low” for diarrhea. First of all, a downgrade was performed due to the relevant risk of bias, mostly related to blinding procedures. Then, we downgraded for inconsistency in the analysis for diarrhea because of the high levels of heterogeneity (I^2^: 90%). There was no inconsistency for IBS symptoms (I^2^: 3%), abdominal pain (I^2^: 19%), and constipation (I^2^: 4%). Lastly, imprecision represented another issue due to the width of the confidence intervals. As known, “low” and “very-low” quality of evidence implies that the real estimate of the effect may be/is very likely to be different from the one obtained in the analysis.

### 4.2. Limitations

This systematic review has some limitations. Even though we included as many languages as possible—English, French, German and Italian—some studies in other languages may have been lost. Additionally, we reached a limited number of studies. The inclusion criteria for the intervention were broad. In fact, no limitations have been imposed on the type of intervention regarding the use of specific techniques, dosage, and duration of treatment. This does not allow for an optimal comparison of the results of the analyzed studies. Similarly, no limits were placed on the selection of the control group. In two studies, only counseling from the therapist was received [30,33], while in the other three it received a sham treatment [29,31,32]. Furthermore, publication bias was not evaluated since no statistical tool is able to detect it [49], therefore, it may be present.

## 5. Conclusions

This first meta-analysis evaluating the effectiveness of OMT for the severity of IBS symptoms highlights the limitations in the number of studies, the low methodological quality of studies, and the heterogeneity of the manipulative techniques used, under the OMT umbrella. For these reasons, despite the physiological basis of osteopathic treatment on the autonomic nervous system, we cannot conclude that OMT is effective in the treatment of adults with IBS based on the fascial and visceral component, which impacts on certain conditions such as gastroenteric function, abdominal pain, and constipation. It is important to emphasize the safety of the OMT in those with IBS, as none of the included studies reported major adverse events. Therefore, based on the neurobiological effects of OMT, and to allow a concrete generalization on IBS, we suggest new double-blind RCTs, with a specificity in osteopathic diagnosis, that can increase the methodological quality.

## Figures and Tables

**Table 1:** Overview of the included studies.

Author/Year	Design	Objective	Outcomes	Population	Intervention and Comparison
Müller et al. (2002) [29]	Parallel RCT	Efficacy of OMT on IBS symptoms	(1) Abdominal pain (VAS 0–100) * (2) Intensity and frequency of flatulence (3) Intensity and frequency of constipation (4) Intensity and frequency of diarrhea	N = 60 Male = 17% Age = 48.64 ± 2.79 Pain = 63.63 ± 2.96 IBS duration: at least 1 year	OMT (n = 29) *Description*: 5 sessions every two weeks; duration up to 45 min. Follow-up at 12 weeks (2 weeks after the last session) CG (n = 24) *Description*: 5 sessions every two weeks of sham therapy; duration up to 30 min
Hundscheid et al. (2007) [30]	Parallel pilot RCT	Efficacy of OMT on IBS symptoms and general well-being	(1) IBSQOL 2000 questionnaire * (2) Symptom Score for abdominal pain, cramps, borborygmi, diarrhea, constipation, meteorism, flatulence, feeling of incomplete evacuation of feces and presence of mucous (5-point Likert scale) * (3) FBDSI (4) AEs	N = 39 Male= 41% Age: 43.75 FBDSI: 172.5	OMT (n = 20) *Description*: 5 sessions once per 2–3 weeks. Follow-up 6 months CG (n = 19) *Description*: standard care
Florance et al. (2012) [31]	Parallel pilot RCT	Efficacy of OMT on IBS severity	(1) IBS severity score * (2) Severity of abdominal pain (3) Bristol Stool Form scale (4) FIS (5) BDI (6) HAD (7) Level of satisfaction (8) AEs	N = 30 Male = 23% Age: 47.5 ± 16.8 Severity of IBS: 287 ± 81.5 IBS duration (years): 11.35 ± 13.85	OMT (n = 20) *Description*: 2 sessions with a 7 day interval; duration each session: 60 min CG (n = 10) *Description*: sham OMT
Attali et al. (2013) [32]	Crossover RCT	Efficacy of visceral OMT on symptoms, colonic transit time and rectal sensitivity in refractory IBS patients	(1) IBS symptoms measured with 10-cm VAS (constipation, diarrhea, abdominal distension, and abdominal pain) * (2) Depression (3) Rectal sensitivity (4) Colonic transit	N = 31 Male = 26% Age: 50 ± 2 Abdominal pain: 6.29 ± 0.47 IBS duration (years): 10 ± 4	OMT (n = 15) *Description:* 3 sessions of OMT separated by 2-week intervals, and 3 sessions of placebo separated by 2-week intervals; duration of each session: 45 min CG (n = 16) *Description:* 3 sessions of placebo separated by 2-week intervals, and 3 sessions of OMT separated by 2-week intervals; duration of each session: 45 min
Piche et al. (2014) [33]	Parallel RCT	Efficacy of OMT on the severity of IBS-like symptoms in quiescent CD patients	(1) IBS severity score * (2) IBDq (3) FIS (4) 13-item BDI questionnaire (5) HAD scale (6) Antispasmodic utilization (7) Level of satisfaction (8) AEs	N = 37 Male = 35% Age: 41.75 (35.1–49) Severity of IBS: 71.5 (45.5–119.0) IBS duration (years): 7.5 (2–12)	OMT (n = 25) *Description*: 3 sessions of OMT at day 15, 30, and 45, after the last infusion of TNF-α (infliximab) at day 0; duration of each session: 60 min CG (n = 12) *Description*: 3 sessions of caring attention and listening without manipulation at day 15, 30 and 45, after the last infusion of TNF-α at day 0
Bouchoucha et al. (2018) [34] NCT02932111	Parallel RCT, ongoing study (estimated completion date: 12 November 2023)	Efficacy of OMT in IBS symptoms	(1) IBS severity score * (2) Cardinal signs of IBS measured with VAS (constipation, diarrhea, abdominal pain, bloating, topology of abdominal pain) (3) Bristol Stool Form scale (4) BDI-II questionnaire (5) IBSQOL (6) STAI	N = 210 Age: ≥18 years Sexes: both	OMT *Description:* 3 sessions of OMT over a 6-week treatment period. CG *Description*: 3 sessions of sham OMT over a 6-week treatment period.

* primary outcome. Abbreviations: RCT “Randomized Controlled Trial”, VAS “Visual Analogue Scale”, OMT “Osteopathic Manipulative Treatment”, IBS “Irritable Bowel Syndrome”, CG “Control Group”, IBSQOL questionnaire “Irritable Bowel Syndrome Quality Of Life questionnaire”, FBDSI “Functional Bowel Disorder Severity Index”, AEs “Adverse Events”, FIS “Fatigue Impact Scale”, BDI questionnaire “Beck Depression Inventory questionnaire”, HAD scale “Hospital Anxiety and Depression scale”, CD “Celiac Disease”, IBDq “Inflammatory Bowel Disease questionnaire”, TNF-α “anti-tumor necrosis factor-α”, STAI “State-Trait Anxiety Inventory”.

**Table 2:** Description of interventions and main results of the included studies.

Author/Year	Description of Interventions	Main Results
Müller et al. (2002) [29]	*OMT group:* specific OMT During each session an osteopath performed an evaluation of the suture occipito mastoidea, epigastric zone, and colon in relation to small intestine and to parietal planes. According to the severity (0–2), the osteopath treated the dysfunctional areas. Each technique was performed for about 8 min. *CG:* sham OMT The patients underwent an osteopathic examination of the spine from T11 to L5, ribs 11 and 12, symphysis, sacroiliac joint and coccygeum. Both groups were allowed to take medications, except during the 48 h before the treatment	***Abdominal pain (VAS)*** *OMT*: baseline—30 days (*p* = 0.0002) baseline—45 days (*p* = 0.0103) baseline—60 days (*p* < 0.0001) baseline—75 days (*p* < 0.0001) *CG*: baseline—75 days (*p* = 0.017) *OMT vs. CG:* at 75 days in favor of OMT (*p* < 0.0001) ***Diarrhea*** *OMT vs. CG:* at 60 days in favor of OMT (*p* = 0.0225) at 75 days in favor of OMT(*p* = 0.0165) ***Constipation*** *OMT vs. CG:* at 60 days in favor of OMT (*p* = 0.0183) at 75 days in favor of OMT (*p* = 0.0080)
Hundscheid et al. (2007) [30]	*OMT group:* black box method OMT was performed adapting the treatment to the clinical history of each patient. Patients could not to take any medication used in the standard care of IBS, and they did not nor receive the advice to consume more fiber. *CG:* standard care Patients were advised to have a diet rich in fiber. They could take extra fiber or laxatives in case of constipation, loperamide for predominant diarrhea, and mebeverine for cramps.	***IBSQOL*** *OMT:* baseline—month 3: from 111 ± 22 to 125 ± 20 (*p* < 0.009) baseline—month 6: from 111 ± 22 to 129 ± 19 (*p* < 0.009) *CG:* no statistically significant differences (*p* > 0.05) ***Symptom score*** *OMT:* no statistically significant differences (*p* > 0.05) *CG:* no statistically significant differences (*p* > 0.05) *OMT vs. CG:* OMT was superior to CG (*p* = 0.02) ***FBDSI*** *OMT:* baseline—month 6: from 174 ± 36 to 74 ± 64 (*p* < 0.0001) *CG:* baseline—month 6: from 171 ± 31 to 119 ± 48 (*p* < 0.0001) *OMT vs. CG:* OMT was superior to CG (*p* = 0.02) ***AEs*** None. *OMT*: slight increase in the severity of the symptoms after the first session which resolved quickly.
Florance et al. (2012) [31]	*OMT group:* standardized OMT During each session an osteopath performed a physical examination and treatment of the spine and the abdomen. The operator used both direct techniques (hand pressure on each segment of the spine for 90 s) and an indirect technique (pressure on the segment using hands, knees, or the chest). Each session was completed by visceral osteopathy. *CG:* sham OMT The osteopath touched the same areas treated in the OMT group (spine and abdomen) with a gentle massage.	***IBS severity score*** *OMT:* baseline—day 7: 196 ± 88 (*p* < 0.01) baseline—day 28: 224 ± 102 (*p* < 0.01) *CG:* baseline—day 7: 244 ± 75 (*p* = 0.04) baseline—day 28: no statistically significant differences (*p* = 0.07). *OMT vs. CG:* at day 7 in favor of OMT (*p* = 0.01) at day 28 no differences (*p* = 0.8). ***Stool frequency and consistency*** No statistically significant differences (*p* > 0.05) ***AEs*** None.
Attali et al. (2013) [32]	*OMT group:* standardized OMT The osteopath performed abdominal and sacral gentle manipulations. At the beginning of the session, the operator applied a global visceral technique, performing gentle vibrations with both hands. Then, the areas addressed as highly sensitive by the patients were treated by pressing and vibrating the fingers. Eventually, the osteopath performed a sacral technique. *CG*: sham OMT Superficial massage in the same points of the OMT. Movements were similar in variety and duration. After 3 sessions of either OMT or sham OMT, the two groups switched to the other intervention: group A received sham OMT in Phase 1 and OMT in Phase 2, while group B received OMT in Phase 1 and sham OMT in Phase 2.	***IBS symptoms*** *Phase 1* *OMT (group B):* baseline—week 5: constipation (*p* = 0.022) diarrhea (*p* = 0.016) abdominal distension (*p* = 0.001) abdominal pain (*p* = 0.005) *CG (group A):* baseline—week 5: abdominal distension (*p* = 0.026) abdominal pain (*p* = 0.001) *OMT vs. CG:* at week 5 no statistically significant differences (*p* > 0.05). *Phase 2* *OMT (group A):* week 5—week 11: abdominal distension (*p* = 0.002) abdominal pain (*p* = 0.003) *CG (group B):* week 5—week 11: no differences (*p* > 0.05) *All patients:* baseline—week 11: constipation (*p* < 0.001) diarrhea (*p* = 0.003) abdominal distension (*p* < 0.001) abdominal pain (*p* < 0.001) baseline—1 year: diarrhea (*p* = 0.029) abdominal distension (*p* = 0.001) abdominal pain (*p* < 0.001)
Piche et al. (2014) [33]	*OMT group:* standardized OMT During each session an osteopath performed a physical examination and treatment of the spine and the abdomen. The operator used both direct techniques (hand pressure on each segment of the spine for 90 s) and an indirect technique (pressure on the segment using hands, knees, or the chest). Each session was completed by visceral osteopathy. *CG:* no intervention The osteopath offered caring attention and listening without manipulation.	***IBS severity score*** *OMT:* baseline—15 days: no statistically significant differences (*p* = 0.3) baseline—30 days: 67 (16–116), *p* = 0.05 baseline—45 days: 50 (12–99), *p* = 0.01 baseline—60 days: 32 (15–106), *p* = 0.01 *CG:* no statistically significant differences (*p* > 0.05) *OMT vs. CG:* at 15 days no statistically significant differences (*p* > 0.05) at 30 days in favor of OMT (*p* = 0.01) at 45 days in favor of OMT (*p* = 0.04) at 60 days in favor of OMT (*p* = 0.05) ***IBDq*** *OMT:* baseline—15 days: no statistically significant differences (*p* = 0.6) baseline—30 days: 200 (177–213), *p* = 0.01 baseline—45 days: 204 (174–216), *p* = 0.01 baseline—60 days: 196 (174–209), *p* = 0.05 *CG:* no statistically significant differences (*p* > 0.05) *OMT vs. CG:* at 15, 30 and 60 days no statistically significant differences (*p* > 0.05) at 45 days in favor of OMT (*p* = 0.05) ***AEs*** None. *OMT:* brief sensation of fatigue immediately after intervention.
Bouchoucha et al. (2018) [34] NCT02932111	*OMT group:* standardized OMT Friction in the hourly sense, vibration, inhibitions or rebounds in the abdominal projection of the junction, where there is the trigger zone. *CG:* sham OMT The osteopath will mimic the techniques provided in the OMT group without any intention of treatment.	Ongoing study, hence no reported results.

Abbreviations: OMT “Osteopathic Manipulative Treatment”, CG “Control Group”, VAS “Visual Analogue Scale”, IBSQOL questionnaire “Irritable Bowel Syndrome Quality Of Life questionnaire”, FBDSI “Functional Bowel Disorder Severity Index”, AEs “Adverse Events”, IBS “Irritable Bowel Syndrome”, IBDq “Inflammatory Bowel Disease questionnaire”, FIS “Fatigue Impact Scale”.

**Table 3:** Quality of evidence assessed through GRADE framework.

Outcome	SMD (95% CI)	N. of Subjects (Studies)	Comments	Quality of Evidence
IBS symptoms (IBS Severity Score and Likert)	−0.34 [−0.83, 0.16]	69 (2 RCT)	Downgraded by 1 level for RoB Downgraded by 1 level for Imprecision	⊕⊕◯◯ **LOW**
Abdominal pain (VAS)	−1.14 [−1.66, −0.62]	84 (2 RCT)	Downgraded by 1 level for RoB Downgraded by 1 level for Imprecision	⊕⊕◯◯ **LOW**
Constipation	−0.66 [−1.12, −0.20]	82 (2 RCT)	Downgraded by 1 level for RoB Downgraded by 1 level for Imprecision	⊕⊕◯◯ **LOW**
Diarrhea	−1.20 [−2.84, 0.43]	81 (2 RCT)	Downgraded by 1 level for RoB Downgraded by 2 levels for Inconsistency (I^2^ = 90%) Downgraded by 1 level for Imprecision	⊕◯◯◯ **VERY LOW**

**High Quality:** We are very confident that the true effect lies close to that of the estimate of the effect. **Moderate quality:** We are moderately confident in the effect estimate; the true effect is likely to be close to the estimate of effect, but there is a possibility that it is substantially different. In this case, three plus are placed in the table. **Low quality:** Our confidence in the effect estimate is limited; the true effect may be substantially different from the estimate of the effect. In this case, two pluls are placed in the table. **Very low quality:** We have very little confidence in the effect estimate; the true effect is likely to be substantially different from the estimate of effect. In this case, one plus is placed in the table.

## Data Availability

Not applicable.

## Appendix A

Research string for each database:

1. **PubMed**

(((((((irritable bowel syndrome[MeSH Terms]) OR (“irritable bowel syndrome*”[Title/Abstract])) OR (constipation[MeSH Terms])) OR (constipation*[Title/Abstract])) OR (“irritable colon”[Title/Abstract])) OR (defecation[MeSH Terms])) OR (defecation*[Title/Abstract]) OR (meteorism[Title/Abstract])) AND ((((((((((((((((manipulation, osteopathic[MeSH Terms]) OR (medicine, osteopathic[MeSH Terms])) OR (osteopat*[Title/Abstract])) OR (“osteopathic treatment*”[Title/Abstract])) OR (“manipulative treatment*”[Title/Abstract])) OR (“osteopathic manipulative treatment*”[Title/Abstract])) OR (“myofascial treatment”[Title/Abstract])) OR (“myofascial therap*”[Title/Abstract])) OR (“myofascial release”[Title/Abstract])) OR (“myofascial technique*”[Title/Abstract])) OR (musculoskeletal manipulations[MeSH Terms])) OR (“musculoskeletal manipulation*”[Title/Abstract])) OR (“craniosacral therap*”[Title/Abstract])) OR (“craniosacral treatment*”[Title/Abstract])) OR (“cranial therap*”[Title/Abstract])) OR (“visceral manipulation*”[Title/Abstract])).

2. **Embase**

(‘irritable colon’:ab,ti OR ‘irritable bowel syndrome’:ab,ti OR constipation:ab,ti OR meteorism:ab,ti) AND (‘osteopathic manipulation’:ab,ti OR ‘osteopathic medicine’:ab,ti OR osteopathic:ab,ti OR ‘myofascial release’:ab,ti OR ‘craniosacral therapy’:ab,ti).

3. **Scopus**

(TITLE-ABS-KEY (irritable AND bowel AND syndrome) OR TITLE-ABS-KEY (irritable AND colon) OR TITLE-ABS-KEY (defecation) OR TITLE-ABS-KEY (constipation) OR TITLE-ABS-KEY (meteorism) AND TITLE-ABS-KEY (osteopathic AND manipulative AND treatment) OR TITLE-ABS-KEY (osteopathic AND manipulation) OR TITLE-ABS-KEY (osteopath*) AND TITLE-ABS-KEY (myofascial AND release) OR TITLE-ABS-KEY (myofascial AND treatment*) OR TITLE-ABS-KEY (craniosacral AND therap*) OR TITLE-ABS-KEY (craniosacral AND treatment*) OR TITLE-ABS-KEY (visceral AND manipulation*) OR TITLE-ABS-KEY (visceral AND therap*) OR TITLE-ABS-KEY (musculoskeletal AND manipulation*) OR TITLE-ABS-KEY (manual AND therap*)).

4. **Cinahl**

(osteopathic manipulative treatment OR osteopathic manipulation OR myofascial release OR myofascial therapy OR craniosacral therapy OR craniosacral treatment OR musculoskeletal manipulations OR visceral manipulation) AND (irritable bowel syndrome OR irritable colon OR constipation OR defecation OR meteorism).

5. **Cochrane**

(“osteopathic manipulative treatment” OR “osteopathic manipulation” OR osteopathic OR osteopathy OR “myofascial release” OR “myofascial therapy” OR “craniosacral therapy” OR “musculoskeletal manipulation”) AND (“irritable bowel syndrome” OR constipation OR defecation OR meteorism) in Title Abstract Keyword.
